# An Evidence-based, Longitudinal Curriculum for Resident Physician Wellness: The 2017 Resident Wellness Consensus Summit

**DOI:** 10.5811/westjem.2017.12.36244

**Published:** 2018-02-26

**Authors:** Jacob Arnold, Jennifer Tango, Ian Walker, Chris Waranch, Joshua McKamie, Zafrina Poonja, Anne Messman

**Affiliations:** * Carl R. Darnall Army Medical Center, Department of Emergency Medicine, Fort Hood, Texas; † Loma Linda University Medical Center, Department of Emergency Medicine, Loma Linda, California; ‡ Sinai-Grace Hospital, Department of Emergency Medicine, Detroit, Michigan; § University of Missouri Hospital, Department of Emergency Medicine, Columbia, Missouri; ¶ Detroit Receiving Hospital, Department of Emergency Medicine, Detroit, Michigan; || University of Alberta, Department of Emergency Medicine, Edmonton, Alberta, Canada

## Abstract

**Introduction:**

Physicians are at much higher risk for burnout, depression, and suicide than their non-medical peers. One of the working groups from the May 2017 Resident Wellness Consensus Summit (RWCS) addressed this issue through the development of a longitudinal residency curriculum to address resident wellness and burnout.

**Methods:**

A 30-person (27 residents, three attending physicians) Wellness Curriculum Development workgroup developed the curriculum in two phases. In the first phase, the workgroup worked asynchronously in the Wellness Think Tank – an online resident community – conducting a literature review to identify 10 core topics. In the second phase, the workgroup expanded to include residents outside the Wellness Think Tank at the live RWCS event to identify gaps in the curriculum. This resulted in an additional seven core topics.

**Results:**

Seventeen foundational topics served as the framework for the longitudinal resident wellness curriculum. The curriculum includes a two-module introduction to wellness; a seven-module “Self-Care Series” focusing on the appropriate structure of wellness activities and everyday necessities that promote physician wellness; a two-module section on physician suicide and self-help; a four-module “Clinical Care Series” focusing on delivering bad news, navigating difficult patient encounters, dealing with difficult consultants and staff members, and debriefing traumatic events in the emergency department; wellness in the workplace; and dealing with medical errors and shame.

**Conclusion:**

The resident wellness curriculum, derived from an evidence-based approach and input of residents from the Wellness Think Tank and the RWCS event, provides a guiding framework for residency programs in emergency medicine and potentially other specialties to improve physician wellness and promote a culture of wellness.

## INTRODUCTION

Many recent academic and popular publications have highlighted the fact that physicians are at much higher risk for burnout, depression, and suicide than the general population of the United States. Data from the National Violent Death Reporting System indicate that each year more than 200 physicians in the U.S. commit suicide.^1^ Medical students and residents are at especially high risk.^2^ Furthermore, emergency physicians are consistently ranked at the top of most burnt-out doctors.^3^ This dark problem was recently brought to the forefront in an email written to the Council of Emergency Medicine Residency Directors by Dr. Christopher Doty, the residency program director at the University of Kentucky, detailing his tragic loss of a resident and its effects on the residency and broader hospital community.

The Accreditation Council for Graduate Medical Education (ACGME) has included the mandate that residency programs address resident wellness within the Common Program Requirements. Emergency medicine (EM) residency programs are now required to provide education to residents and faculty on burnout, depression and substance abuse and are instructed to implement curricula to encourage optimal wellbeing. In 2016 a group of 142 EM residents from across the world began discussing ways to address this issue through the Wellness Think Tank, a virtual community of practice focusing on resident wellness. Members shared personal stories of hardship, identified obstacles to wellness, and brainstormed solutions to this multifaceted problem. This online collaboration also served as the virtual platform to asynchronously collaborate on pre-work for the inaugural Resident Wellness Consensus Summit (RWCS) in Las Vegas, NV, on May 15, 2017.^4^ One of the working groups was to develop a structured, longitudinal, residency curriculum based on the existing literature to address resident wellness and burnout. Herein we report the consensus recommendation of the Wellness Curriculum Development working group.

## METHODS

The wellness curriculum was developed in two phases. During the first phase, members of the Wellness Curriculum Development working group in the Wellness Think Tank collectively performed an extensive literature search, targeting articles that focused on resident wellness, physician wellness, and previous wellness initiatives. These articles were divided up among the group members and carefully evaluated. They found 21 relevant articles. Individual curricular initiatives were categorized by theme.^1^–^3^,^5^–^7^,^9^–^23^ These themes informed the initial framework of 10 core topics for the curriculum. For each topic, a sub-team conducted a deeper analysis before providing a description of the module, recommended approach, and additional resources and recommended readings.

The second phase of curriculum development occurred at the RWCS event. Members of the Wellness Curriculum Development workgroup presented the proposed curriculum to other summit attendees for feedback and further evaluation. This working group had a total of 30 members, 27 residents and three attending physicians. The resident cohort of this group specifically contained 15 Wellness Think Tank members, an additional 12 non-Think Tank residents. At the summit, there was consensus that the initial 10 topics were necessary components for the curriculum. Gaps, however, were identified and through further discussion, an additional seven topics were added. Each topic was subsequently reviewed after the RWCS for a final total of 17 modules for the resident physician wellness curriculum.

## RESULTS

After a literature search and the RWCS event, we identified 17 foundational topics to incorporate into the resident physician wellness curriculum (Table). Topics were chosen that contribute to personal wellness, with a focus specifically for the EM resident. All of these topics are intended to consist of a short, large-group lecture and small-group breakout sessions. The full curriculum resource, which includes relevant technologies from the Wellness Technologies working group, is outlined in the Appendix.

### Introduction

The curriculum is meant to launch at the start of the academic year. The first module, “Introduction to Wellness,” focuses on the definition of wellness, a preview of the different components of the curriculum, and what residents should ideally gain from the curriculum. The following “Why Wellness Matters” module examines the concept of burnout and the associated statistics. During this portion of the curriculum, residency training programs should conduct burnout screening for each resident.

### Self-Care Series

This seven-part series focuses on everyday necessities that promote physician self-care and wellness. It begins with a qualitative look at wellness activities that physicians perform to maintain their overall wellbeing. This is supported by current literature showing the priority of activities that physicians who display wellness find most important. These include sleep, nutrition, physical fitness, financial health, mindfulness, and having a support network. Specifically for the mindfulness module, another RWCS workgroup created educator toolkits on positive psychology and mindfulness/meditation, which can be incorporated in this section.^4^ Each module further delves into specific challenges unique to emergency physicians.

### Physician Suicide

This two-module block focuses on the facts and realities of physician suicide. When age-matched with peers from other professions, physicians are at significant risk for suicide.^5^ These modules open the discussion on screening for physician suicide for others and oneself, possible resources for intervention, and long-term follow up. Importantly it also provides more exposure to this topic to help normalize the conversation in a respectful and psychologically safe environment.^6^

### Clinical Care Series

This four-part series focuses on the following high-stress activities that resident physicians encounter in the emergency department (ED): delivering bad news, managing difficult patient encounters, managing difficult consultants and staff members, and debriefing traumatic events. Because delivering bad news to patients and their families can produce high levels of real-time and ongoing anxiety for the resident, one module focuses on these stressful conversations. A framework is provided to help them navigate these conversations. Difficult patient encounters can also put undue strain onto the emergency physician. One module thus focuses on dealing with difficult patients, specifically identifying triggers and creating preformed responses while also maintaining physician empathy and the physician-patient relationship.

Such preparation often leads to better patient care and less physician burnout.^7^ In the same way that a difficult patient can lead to decreased physician happiness and satisfaction, so can a bad interaction with a consultant or staff member. This module focuses on practical strategies to keep these encounters professional, positive, and effective. The last topic in this series is debriefing traumatic events that occur in the ED. Practical tips are outlined to overcome many of the barriers to conduct these guided group reflections. Within this last module, one might incorporate a discussion of the second victim syndrome, which is an educator toolkit developed by one of the RWCS workgroups.^4^ This phenomenon, whereby a healthcare provider is traumatized by an unanticipated, adverse, patient-related event, is an important but often under-recognized problem facing emergency physicians.

### Miscellaneous Topics

The last two modules included are no less important. The first addresses the wellness culture within the workplace. Basic on-shift physician wellness needs (e.g., bathroom break, meal, snack) rely on a supportive culture. It should not be viewed as a sign of weakness to take care of one’s basic human needs. Not caring for oneself will ultimately hamper patient care at a time when patients need us working at our very best.

The second module focuses on dealing with medical errors and shame. To err is human, and all physicians will make mistakes. Dealing with the natural feelings of inadequacy and shame in a healthy and constructive manner promotes learning and growth, rather than self-destructive responses and harmful behaviors.

## DISCUSSION

The term “physician wellness” has many definitions, and might best be defined as “one’s personal recipe for thriving” and not just surviving.^8^ It is not, however, merely the absence of burnout, depression, or suicide. Teaching this concept during residency training is an ideal time to address physician wellness. This is especially crucial for EM residents, because EM as a specialty has the highest rate of burnout per the Maslach Burnout Inventory.^3^ These new physicians can develop healthy mindset practices, coping skills, and work-life balance habits that they will use throughout their careers.

The proposed 17-topic wellness curriculum focuses on the spectrums of wellness and burnout in a modular fashion, as framed by the existing literature. Based on residency program needs, these modules can be rearranged. Alternatively, suggested materials from some/all of the modules can be emailed to residents to serve as self-study resources.

One study demonstrated that discussing and reflecting on wellness topics in small groups has positive downstream effects. West et al. performed a randomized clinical trial in which all participating physicians were given paid time off to work on aspects of wellness.^9^ The intervention arm met in a small group for one hour every two weeks to discuss wellness topics, while the control arm had no formal intervention. The study found that empowerment and engagement at work significantly increased in the intervention arm, and decreased in the control arm. They also found that rates of overall burnout, emotional exhaustion, and depersonalization in the intervention arm dropped substantially and only decreased slightly in the control arm. Thus, a formal wellness curriculum during residency training, if done well, has the potential to make a lasting positive impact on resident wellness. The recent mandate from ACGME to address resident wellness in the Common Program requirements is an important step towards improving resident wellness. Ultimately, a multi-pronged approach toward improving resident wellness will be needed and must include systemic changes in order to reach its full potential.

## CONCLUSION

In the past few years, much light has been shed on the colossal topic of wellness, specifically that physicians are at a high risk of suicide and emergency physicians rank highest for physician burnout.^1^,^3^ The RWCS event was created to address this issue specifically at the level of graduate medical education for EM residents. Through pre-work by the Wellness Think Tank community and consensus discussions at the live RWCS event in the Wellness Curriculum Development workgroup, an evidence-based, 17-module, longitudinal, wellness curriculum was designed for EM residency programs. Many of these modules may be applicable for residency programs in other specialties, as well as the broader physician community. As we receive feedback from residency programs, we hope to continually revise and reshape the curriculum with the overarching goal of helping to advance the culture of wellness during residency training beyond one of survival to one of thriving.

## Supplementary Information



## Figures and Tables

**Table:** A longitudinal 17-module physician wellness curriculum for emergency medicine residents.

Topic	Description
1. Introduction to Wellness	Introduction to physician wellness and burnout, the longitudinal wellness curriculum, and a breakout session for interns on “Transitioning to Residency”
2. Why Wellness Matters	Building awareness on burnout, depression and mental health issues in physicians, and that wellness is not the absence of distress
3. Self-Care Series: Wellness Activities of Physicians	A discussion of how physicians stay well through relationships, religion and spirituality, self-care, work, and approach to life
4. Self-Care Series: Sleep	Education on sleep hygiene, scheduling, and practical tips for the shift-based emergency physician life
5. Self-Care Series: Nutrition	Education on the basics of nutrition and how to eat a healthy, balanced diet, particularly for people with busy lifestyles
6. Self-Care Series: Physical Fitness	Education on the basics and scientifically proven benefits of physical fitness, as well as how to get started on an exercise program
7. Self-Care Series: Financial Health	Overview of the basics of budgeting, living within your means, and tackling student loan debt; breakout session recommended specifically for graduating senior residents
8. Self-Care Series: Mindfulness and Reflection	Overview of the concept, scientifically-proven benefits, unwarranted stigma, and practice of mindfulness for the busy resident physician
9. Self-Care Series: Building Your Support Network	Discussion about the importance of a support network for the resident, especially a mentorship program, in promoting wellness and building resiliency
10. Physician Suicide	Education on risk factors for depression and suicide specific to physicians, and how to recognize them in yourself
11. “I Need Help”	Education on how to get mental health help for oneself, with a focus on systems that ensure confidentiality
12. Clinical Care Series: Delivering Bad News	Education for resident physicians on how to deliver bad news to patients and their families
13. Clinical Care Series: Dealing with Difficult Patients	Education on how to appropriately manage difficult patient encounters with evidence-based recommendations for success
14. Clinical Care Series: Dealing with Difficult Consultants and Staff	Education on how to appropriately and professionally interact with difficult consultants and staff members
15. Clinical Care Series: Debriefing Traumatic Events in the Emergency Department	Education about debriefing techniques following significant events in the emergency department to ensure a collective, safe, guided reflection of the event
16. Wellness in the Workplace	Discussion about how individual wellness depends on the supportive workplace wellness culture
17. Dealing with Medical Errors and Shame	Education on how residents can cope with medical errors in a healthy fashion to minimize feelings of inadequacy, shame, and burnout
