# Supplementary material for: An Evidence-based, Longitudinal Curriculum for Resident Physician Wellness: The 2017 Resident Wellness Consensus Summit
Source: West J Emerg Med. 2018 Feb 26;19(2):337–41. doi: 10.5811/westjem.2017.12.36244 (PMC5851508; doi:10.5811/westjem.2017.12.36244)
Supplement: Supplementary file 1 [file wjem-19-337-s001.pdf]

An Evidence-Based, Longitudinal Curriculum for Resident Physician Wellness

2017 Resident Wellness Consensus Summit

Arnold J, Tango J, Walker I, Waranch C, McKamie J, Poonja Z, Messman A
Appendix to publication: … *[insert WestJEM citation]*

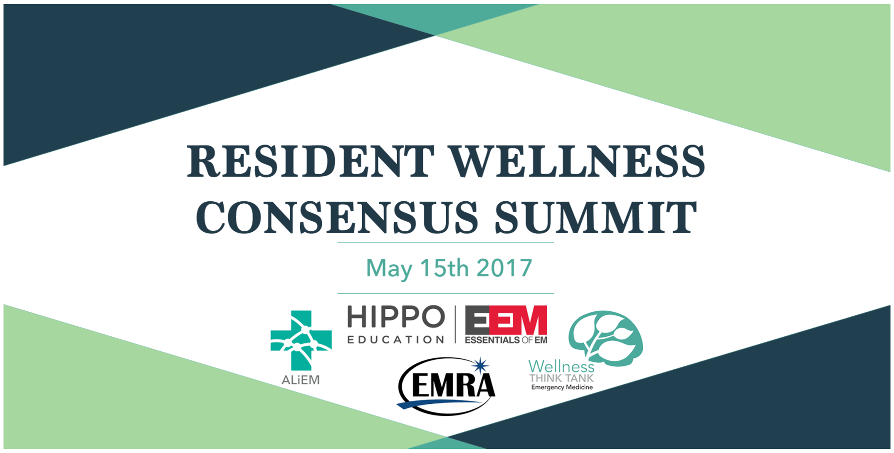

Table of Contents

Topic	Description	Pages
1. **Introduction to Wellness**	Introduction to physician wellness and burnout, the longitudinal wellness curriculum, and a companion guide for interns on "Transitioning to Residency"	4-5
2. **Why Wellness Matters**	Building awareness on burnout, depression and mental health issues in physicians, and that wellness is not the absence of distress	6-7
3. Self-Care Series: **Wellness Activities of Physicians**	A discussion of how physicians stay well through relationships, religion and spirituality, self-care, work, and approach to life	8
4. Self-Care Series: **Sleep**	Education on sleep hygiene, scheduling, and practical tips for the shift-based emergency physician life	9-10
5. Self-Care Series: **Nutrition**	Education on the basics of nutrition and how to eat a healthy, balanced diet, particularly for people with busy lifestyles	11-12
6. Self-Care Series: **Physical Fitness**	Education on the basics and scientifically proven benefits of physical fitness as well as how to get started on an exercise program	13-14
7. Self-Care Series: **Financial Health**	Overview of the basics of budgeting, living within your means, and tackling student loan debt; companion guide included specifically for graduating senior residents	15-16
8. Self-Care Series: **Mindfulness and Reflection**	Overview of the concept, scientifically proven benefits, unwarranted stigma, and practice of mindfulness for the busy resident physician	17-18
9. Self-Care Series: **Building Your Support Network**	Discussion about the importance of a support network for the resident, especially a mentorship program, in promoting wellness and building resiliency	19-20

10. **Physician Suicide**	Education on risk factors for depression and suicide specific to physicians, and how to recognize them in yourself	21
11. **"I Need Help"**	Education on how to get mental health help with a focus on systems that ensure confidentiality	22-23
12. Clinical Care Series: **Delivering Bad News**	Education for resident physicians on how to deliver bad news to patients and their families.	24-25
13. Clinical Care Series: **Dealing with Difficult Patients**	Education on how to appropriately manage difficult patient encounters with evidence-based recommendations for success	26-27
14. Clinical Care Series: **Dealing with Difficult Consultants and Staff**	Education on how to appropriately and professionally interact with difficult consultants and staff members	28-29
15. Clinical Care Series: **Debriefing Traumatic Events in the Emergency Department**	Education about debriefing techniques following significant events in the Emergency Department to ensure a collective, safe, guided reflection of the event	30-31
16. **Wellness in the Workplace**	Discussion about how individual wellness depends on the supportive workplace wellness culture	32-33
17. **Dealing with Medical Errors and Shame**	Education on how residents can cope with medical errors in a healthy fashion to minimize feelings of inadequacy, shame, and burnout	34-35

1. Introduction to Wellness

Objective

Introduce the concepts of wellness and burnout, with a breakout session specifically for new interns who are transitioning from medical school to residency

Recommended Approach

Start by discussing what wellness is to you (as a resident or faculty member), and why is it so important. This could be related to a personal anecdote, hearing about other resident struggles, or discussing the common challenges of residency and how they affect all residents.

After this audience participation and engagement section, the remainder of the session should focus more on the science of wellness. Define wellness, discuss the different elements of wellness), discuss the importance of wellness quoting rates of burnout, depression, suicide, justify why this curriculum is necessary. To re-engage the audience, inquire what "wellness" means to them. The session should conclude by previewing the different components of the upcoming longitudinal curriculum.

Breakout Session: Transition to Residency for New Interns

It is important to include a discussion of wellness during intern orientation. Similar to the introduction, this should be focused on introducing the incoming intern class to the concept of wellness and real risks of burnout. This should include providing a list of resources and support options offered by the program.

Recommended Reading

1. Brazeau CM, Shanafelt T, Durning SJ, et al. Distress among matriculating medical students relative to the general population. Acad Med. 2014 Nov;89(11):1520-5. PMID 25250752
2. Ishak WW, Lederer S, Mandili C, et al. Burnout during residency training: a literature review. J Grad Med Educ. 2009 Dec;1(2):236-42. PMID 21975985
3. Salles A, Liebert CA, Greco RS. Promoting Balance in the Lives of Resident Physicians: A Call to Action. JAMA Surg. 2015 Jul;150(7):607-8. PMID 25992632
4. Sen S, Kranzler HR, Krystal JH, et al. A prospective cohort study investigating factors associated with depression during medical internship. Arch Gen Psychiatry. 2010 Jun;67(6):557-65. PMID 20368500
5. Shanafelt TD, Hasan O, Dyrbye LN, et al. Changes in Burnout and Satisfaction With Work-Life Balance in Physicians and the General US Working Population Between 2011 and 2014. Mayo Clin Proc. 2015 Dec;90(12):1600-13. PMID 26653297
6. West CP, Dyrbye LN, Rabatin JT, et al. Intervention to promote physician well-being, job satisfaction, and professionalism: a randomized clinical trial. JAMA Intern Med. 2014 Apr;174(4):527-33. PMID 24515493

Additional Resources

None

2. Why Wellness Matters

Objective

Provide awareness of burnout, depression, and mental health issues in physicians, with a specific notion that wellness is not the absence of distress.

Recommended Approach

In a large group setting, review the statistics or burnout in the emergency medicine profession. In a large or small group setting, discuss actual examples of those who were affected by depression. Encourage testimonials from staff members and residents. Discuss the importance of having hobbies and non-medical interests, and share what one's escape is during stressful times.

In this session, disseminate a wellness or burnout assessment tool (e.g. Maslach Burnout Inventory, Life Orientation Test-Revised) to the residents assess burnout and those at risk.

Recommended Reading

1. Dyrbye LN, West CP, Satele D, Boone S, Tan L, Sloan J, Shanafelt TD. Burnout among U.S. medical students, residents, and early career physicians relative to the general U.S. population. Acad Med. 2014 Mar;89(3):443-51. PMID 24448053
2. Levey RE. Sources of stress for residents and recommendations for programs to assist them. Acad Med. 2001 Feb;76(2):142-50. PMID 11158832
3. Rubin R. Recent suicides highlight need to address depression in medical students and residents. JAMA. 2014 Nov 5;312(17):1725-7. PMID 25369478
4. Shanafelt TD, Sloan JA, Habermann TM. The well-being of physicians. Am J Med. 2003 Apr 15;114(6):513-9. PMID 12727590
5. Wallace JE, Lemaire JB, Ghali WA. Physician wellness: a missing quality indicator. Lancet. 2009 Nov 14;374(9702):1714-21. PMID 19914516

Additional Resources

1. Herbert M. The Dark Side of EM. Emergency Medicine: Reviews and Perspectives (EM:RAP) podcast. https://www.emrap.org/episode/burnout/burnout. April 1, 2017. Accessed August 6, 2017.
2. Khan R. This doctor beat burnout by doing these 5 things. KevinMD.com website. http://www.kevinmd.com/blog/2017/03/doctor-beat-burnout-5-things.html. March 28, 2017. Accessed August 6, 2017.
3. Konopasek L, Bernstein C. Combating Burn Out, Promoting Physician Well-Being: Building Blocks for a Healthy Learning Environment in GME. Accreditation Council for Graduate Medical Education website. https://www.acgme.org/Portals/0/PDFs/Webinars/July_13_Powerpoint.pdf. July 13, 2016. Accessed August 6, 2017.
4. Mini Z burnout survey. AMA Steps Forward website. https://www.stepsforward.org/modules/physician-burnout-survey. Accessed August 6, 2017.

Self-Care Series

3. Wellness Activities of Physicians

Objective

Discuss how physicians stay well, specifically with regards to relationships, religion/spirituality, self-care, work, and approach to life.

Recommended Approach

A short talk led by a faculty member on their personal experience maintaining work-life balance, followed by group discussion on the topic. Throughout the talk, encourage other faculty and residents to share their own strategies with the audience.

Recommended Reading

- Weiner EL, Swain GR, Wolf B, Gottlieb M. A qualitative study of physicians' own wellness-promotion practices. West J Med. 2001 Jan;174(1):19-23. PMID 11154656

Additional Resources

None

Self-Care Series

4. Sleep

Objective

Provide evidence-based education on sleep hygiene and scheduling, and discuss how to improve sleep quality in a 24/7 shift-based profession.

Background Information

As emergency physicians, we pride ourselves on taking care of patients 24 hours a day. Managing the inevitable sleep disturbances is essential to a successful career and happy life. This is especially important during residency, when there are typically more irregular and frequent night shifts, in addition to off-service rotations with 24+ hour calls.

Recommended Approach

In a large group setting, this didactic session should review the basic science behind circadian rhythms, the dangerous effects of sleep deprivation and irregular schedules, and the various coping strategies. The majority of the session should be spent on optimizing sleep hygiene, the dangers of relying on chemical substances (benzodiazepines, alcohol, caffeine), and sharing successful practical strategies from the audience.

Recommended Reading

1. Eastman CI, Stewart KT, Mahoney MP, et al. Dark goggles and bright light improve circadian rhythm adaptation to night-shift work. Sleep. 1994 Sep;17(6):535-43. PMID 7809567
2. Kuhn G. Circadian rhythm, shift work, and emergency medicine. Ann Emerg Med. 2001 Jan;37(1):88-98. PMID 11145778

Additional Resources

1. Pzziz app: An Android and iPhone app to help you take a great "power nap" or get to sleep at night. http://pzizz.com/.
2. Schley C. The Best White Noise Machine. The Sweet Home website. http://thesweethome.com/reviews/best-white-noise-machine/. March 27, 2017. Accessed August 6, 2017.
3. Additional relevant apps as reviewed by the Resident Wellness Consensus Summit working group on Best Wellness Technologies:
   - Sleep Cycle: https://www.sleepcycle.com/
   - Bellabeat: https://www.bellabeat.com/urban
   - Rain Rain: https://www.rainrainapp.com/
   - Deep Sleep: https://itunes.apple.com/us/app/deep-sleep-andrew-johnson/id337349999?mt=8
   - f.lux: https://justgetflux.com/
   - Power Nap: https://itunes.apple.com/us/app/power-nap-app-best-napping-timer-for-naps-relaxing/id866001468?mt=8

Self-Care Series

5. Nutrition

Objective

Provide evidence-based education on the science of nutrition and how to eat a healthy, balanced diet especially for those with busy lifestyles.

Recommended Approach

In a large group setting, this didactic session should review the science of nutrition, which residents rarely are taught about during residency or even medical school. Explain how to eat for performance and body composition goals, such as eating to gain muscle, lose fat, and perform better. Engage the residents and faculty by having them share how they each incorporate nutrition into their daily busy lives. This may include meal preparation techniques and calorie counting through various digital platforms. Ultimately nutrition goes hand-in-hand with the upcoming session on physical fitness.

Recommended Reading

1. Brewster J. Beginning Nutrition: The Facts About Protein, Carbs & Fat. Bodybuilding.com website. https://www.bodybuilding.com/content/beginning-nutrition-the-facts-about-protein-carbs-fat.html. January 1, 2016. Accessed August 6, 2017.
2. Nutrition. Biolayne website. https://www.biolayne.com/articles/nutrition/. Accessed August 6, 2017.
3. Physician Resources. Physicians Committee for Responsible Medicine website. http://www.pcrm.org/for_physicians. Accessed August 6, 2017.

Additional Resources

1. IIFYM Calculator. If It Fits Your Macros website. https://www.iifym.com/iifym-calculator/. Accessed August 6, 2017.
2. Additional relevant technologies as reviewed by the Resident Wellness Consensus Summit working group on Wellness Technologies:
   - Calorie counting online platform
     - My Fitness Pal: https://www.myfitnesspal.com/
     - Fat Secret: https://www.fatsecret.com/
   - Apps
     - Noom Coach: https://itunes.apple.com/us/app/noom-coach-healthy-weight-loss-diabetes-more/id634598719?mt=8
     - Lifesum: https://www.lifesum.com/

Self-Care Series
6. Physical Fitness

Objective

Provide an evidence-based education on the basics of physical fitness and tips on how to get started on an exercise program.

Recommended Approach

In this large group session, an overview of exercise programs and body mechanics is provided. This may include the following:

- Basics of lifting weights with proper form
- Definitions (such as "sets" and "reps") and structures of workout programs
- Types of cardiovascular exercises
- Resources on beginning and sustaining a workout program

If you have a resident or faculty member, who is educated in physical fitness, encourage taking a group class together. The key is to identify one's ideal exercise plan and stick with it!

Recommended Reading

1. Find a plan: Free fitness programs by the world's best trainers. Bodybuilding.com website. https://www.bodybuilding.com/fun/find-a-plan.html. Accessed August 6, 2017.
2. Klika B, Jordan C. High-Intensity Circuit Training Using Body Weight: Maximum Results With Minimal Investment. *ACSM Health & Fitness Journal.* 2013 May;17(3):8-13. http://journals.lww.com/acsm-healthfitness/Fulltext/2013/05000/HIGH_INTENSITY_CIRCUIT_TRAINING_USING_BODY_WEIGHT_.5.aspx
3. Zamora D. Fitness 101: The absolute beginner's guide to exercise. WebMD website. http://www.webmd.com/fitness-exercise/features/fitness-beginners-guide. Accessed August 6, 2017.

Additional Resources

1. Metzl J, Barrow K. New York Times website. The 9-Minute Strength Workup. https://www.nytimes.com/well/guides/strength-training-plyometrics. Accessed August 6, 2017.
2. Reynolds G. The Scientific 7-Minute Workout. New York Times website. https://www.nytimes.com/interactive/projects/well/workouts/. Accessed August 6, 2017.
3. Additional relevant technologies as reviewed by the Resident Wellness Consensus Summit working group on Wellness Technologies:
   - Sworkit: https://sworkit.com/
   - Yonder: https://www.yonder.it/
   - Zombies Run! https://zombiesrungame.com/
   - Spotify Running: https://www.spotify.com/us/running/
   - Nike+ Training Club: https://www.nike.com/us/en_us/c/nike-plus/training-app
   - Strava: https://www.strava.com/
   - BodySpace: https://bodyspace.bodybuilding.com/
   - Pocket Yoga: http://www.pocketyoga.com/
4. Search YouTube for specific goals for ideas, resources, and motivation.

Self-Care Series

7. Financial Health

Objective

Provide an overview of the basics of budgeting, living within one’s means, and tackling student loan debt.

Recommended Approach

Using the advice of Dr. James Dahle (author of *The White Coat Investor* book) as a guide, introduce residents to the basics of finances. Early education on this subject is important in long-term financial security. Topics to cover should include:

- Budgeting as a resident
- Obtaining disability insurance
- Enrolling for the right student loan repayment plan
- Opening a Roth IRA and/or 401k account
- Basics of how emergency physicians are paid (coding, billing, EM group structures)

Breakout Session: Graduating Senior Residents

In this breakout session, graduating resident should receive more specific information about what to look for in a new job, paying down medical school debt, and living within one’s means until better financial stability is achieved.

Recommended Reading

1. Dahle J. The White Coat Investor: A Doctor's Guide to Personal Finance and Investing. White Coat Investor LLC, 2014. [http://amzn.to/2vvM8Fn].
2. Dahle J. The Five Big Money Items You Should Do as a Resident. White Coat Investor website. https://www.whitecoatinvestor.com/the-five-big-money-items-you-should-do-as-a-resident/. July 7, 2011. Accessed August 6, 2017.

Additional Resources

1. Tracking your money: Mint.com
2. Budgeting: YouNeedABudget.com
3. Automated investing and setting up a Roth IRA: Betterment.com

Self-Care Series

8. Mindfulness and Reflection

Objective

Provide an overview of mindfulness, evidence and ideas on how it can be useful, and examples on how to practice it.

Recommended Approach

In a large group session, introduce the concept of mindfulness and remaining transparent about the initial skepticism it invokes amongst healthcare providers and much of the lay public. Ideally this brief talk would be conducted by a respected and reputable faculty member. The discussion should focus on the scientifically proven benefits of mindfulness and the ease with which it can be practiced. The book *10% Happier*, written by Dan Harris, also features a website and app with introductory content that is well-suited for professionals who may be skeptical of mindfulness.

Following this talk, conduct a brief group activity to demonstrate how little time and effort is needed to practice mindfulness.

Recommended Reading

1. Allen NB, Chambers R, Knight W; Melbourne Academic Mindfulness Interest Group. Mindfulness-based psychotherapies: a review of conceptual foundations, empirical evidence and practical considerations. Aust N Z J Psychiatry. 2006 Apr;40(4):285-94. PMID 16620310
2. Davis DM, Hayes JA. What are the benefits of mindfulness? A practice review of psychotherapy-related research. Psychotherapy (Chic). 2011 Jun;48(2):198-208. PMID 21639664
3. Harris D. 10% Happier: How I Tamed the Voice in My Head, Reduced Stress Without Losing My Edge, and Found Self-Help That Actually Works--A True Story. Dey Street Books, 2014. [http://amzn.to/2flvhPf]
4. Hölzel BK, Carmody J, Vangel M, et al. Mindfulness practice leads to increases in regional brain gray matter density. Psychiatry Res. 2011 Jan 30;191(1):36-43. PMID 21071182
5. Hülsheger UR, Alberts HJ, Feinholdt A, Lang JW. Benefits of mindfulness at work: the role of mindfulness in emotion regulation, emotional exhaustion, and job satisfaction. J Appl Psychol. 2013 Mar;98(2):310-25. PMID 23276118

Additional Resources

1. Dan Harris's website companion to his book "10% Happier" http://www.10percenthappier.com/mindfulness-meditation-the-basics/. Accessed August 6, 2017.
2. Harris D. Meditation 101: A Beginner's Guide (video). Vimeo website. https://vimeo.com/gobblynne/meditation. Accessed August 6, 2017.
3. Additional relevant technologies as reviewed by the Resident Wellness Consensus Summit working group on Wellness Technologies:
   - Daylio: http://daylio.webflow.io/
   - Personal Zen: https://www.personalzen.com/
   - Head Space: https://www.headspace.com
   - Calm: https://www.calm.com/
   - My Mood: https://itunes.apple.com/us/app/my-mood-app/id468442969?mt=8
   - Pacifica: https://www.thinkpacifica.com/
   - Insight Timer: https://insighttimer.com/
   - H*nest Meditation: https://itunes.apple.com/us/app/h-nest-meditation/id1059195733?mt=8

Self-Care Series

9. Building Your Support Network

Objective

Provide advice and tips on building one's own support network to help maintain wellness and resiliency throughout residency training.

Background Information

Having a support network is an essential part of building resiliency in residency and contributes significantly to wellness. This network should ideally be diverse and include not only family members, co-residents, and friends within and outside of medicine, but also mentors who have experienced and successfully navigated the challenges of residency. Mentors can be senior residents and/or practicing physicians.

Ideally the concept of building a support network should have been introduced during intern orientation. Throughout residency (and the rest of their careers), residents should be encouraged to find mentors with interests that align with their own and be given opportunities to meet multiple potential mentors. Mentoring can occur in a one-on-one relationship or in a group fashion. An example of group mentoring might be where a faculty member with specific interests, such as ultrasound, research, or emergency medical services, holds informal resident gatherings quarterly for those interested in that particular area of expertise. All mentor-mentee relationships require maintenance and finding the time talk and meet can be a challenge.

Recommended Approach

In this large or small group session, the concept of mentorship and support networks should be reviewed. Specifically multiple mentors can serve different purposes to help residents reflect on unexpected events and maintain resiliency during residency. Faculty members can share who their mentors are and how their "personal board of directors" (mentorship panel) have evolved over the years.

Programs should consider designating one day every quarter for each resident to focus on growing and maintaining his/her support network.

Recommended Reading

1. Osborn TM, Waeckerle JF, Perina D, Keyes LE. Mentorship: through the looking glass into our future. Ann Emerg Med. 1999 Aug;34(2):285-9. PMID 10424938
2. Yeung M, Nuth J, Stiell IG. Mentoring in emergency medicine: the art and the evidence. CJEM. 2010 Mar;12(2):143-9. PMID 20219162

Additional Resources

1. Academy for Women in Academic Emergency Medicine: Mentorship. Society of Academic Emergency Medicine website. http://www.saem.org/awaem/resources/directories/find-mentorship. Accessed August 6, 2017.
2. Doximity: https://www.doximity.com
3. LinkedIn: https://www.linkedin.com
4. Meetup: https://www.meetup.com

10. Physician Suicide

Objective

Educate residents on suicide risk factors specific to physicians; provide tips on recognizing risk factors for depression and suicide in oneself and others.

Recommended Approach

In a large group didactic, review the statistics that physicians are at a much higher risk of suicide when compared to peers in other professions. Also show statistics on how many medical students, residents, and attending physicians are affected by depression and suicidal ideation. In a large or small group session, have a discussion about the suicide risk factors specific to physicians, and how to recognize these risk factors in themselves and others.

Recommended Reading

1. Downs N, Feng W, Kirby B, et al. Listening to depression and suicide risk in medical students: the Healer Education Assessment and Referral (HEAR) Program. Acad Psychiatry. 2014 Oct;38(5):547-53. PMID 24705825
2. Gold KJ, Sen A, Schwenk TL. Details on suicide among US physicians: data from the National Violent Death Reporting System. Gen Hosp Psychiatry. 2013 Jan-Feb;35(1):45-9. PMID 23123101

11. "I Need Help"

Objective

Educate residents on how to confidentially get help with mental health issues during training.

Recommended Approach

In a large group session, present statistics that show the rates of depression and suicidal ideation in our profession. Discussing this topic openly (and ideally having faculty share their own experiences or stories) helps to remove the stigma around depression and suicidal ideation. Provide residents with institutional resources locally and review issues of confidentiality when seeking help form these resources.

An example of a best-practice program to address depression and suicide amongst learners is the Healer Education Assessment and Referral (HEAR) program at the University of California-San Diego [2]. Discuss amongst the residents and residency leadership to determine whether something similar is feasible for residents locally.

At the conclusion of the session, provide a self-screening survey to each resident to assess his/her own wellness and mental health. Residents should self-score their responses and encourage anyone with concerning scores seek early help and counseling. Preface the survey that such screening tools actually identify a significant number of people who are at early risk, and that there is no shame on or stigma with concerning scores.

Recommended Reading

1. Center C, Davis M, Detre T, et al. Confronting depression and suicide in physicians: a consensus statement. JAMA. 2003 Jun 18;289(23):3161-6. PMID 12813122
2. Downs N, Feng W, Kirby B, et al. Listening to depression and suicide risk in medical students: the Healer Education Assessment and Referral (HEAR) Program. Acad Psychiatry. 2014 Oct;38(5):547-53. PMID 24705825
3. Ey S, Moffit M, Kinzie JM, et al. "If you build it, they will come": attitudes of medical residents and fellows about seeking services in a resident wellness program. J Grad Med Educ. 2013 Sep;5(3):486-92. PMID 24404315
4. Moutier C, Norcross W, Jong P, et al. The suicide prevention and depression awareness program at the University of California, San Diego School of Medicine. Acad Med. 2012 Mar;87(3):320-6. PMID 22373625
5. Williams D, Tricomi G, Gupta J, Janise A. Efficacy of burnout interventions in the medical education pipeline. Acad Psychiatry. 2015 Feb;39(1):47-54. PMID 25034955

Additional Resources

- Interactive Screening Program. American Foundation for Suicidal Prevention website. https://afsp.org/our-work/interactive-screening-program. Accessed August 6, 2017.

Clinical Care Series

12. Delivering Bad News

Objective

Provide education on how to deliver bad news to patients and their families.

Background Information

Delivering bad news is a difficult skill to master. It is not only traumatic to the patients and their families, but oftentimes to the clinician as well, which can negatively impact resident wellness. It is thus important for residents to be trained on thoughtful and clear approaches on delivering bad news. Based on patient surveys, most patients want to know as much as possible especially of the diagnosis and prognosis, but are most concerned with the near-future next steps. There are different approaches in delivering bad news, which include SPIKES, BREAKS, patient and family centered approach, and emotional approach.

SPIKES

1. Set up interviews
2. Assess patient Perspective
3. Obtain patient's Invitation (how much do they want to know)
4. Give Knowledge and information
5. Address Emotions with Empathy
6. Strategy and Summary (have a clear plan for the near future)

BREAKS

1. Background
2. Rapport
3. Exploring
4. Announce
5. Kindling
6. Summarize

It is generally better to use the "**patient and family centered approach**", which involves the family along with the patient during discussion of the bad news. This maintains the patient and patient's needs as the central focus. It leaves the patient feeling hopeful and further trusting of the physician. In contrast, the "**emotional approach**" focuses on the sadness of the message and often leaves the patient feeling sad and hopeless.

Recommended Approach

In a large group setting, discuss the importance of delivering bad news with empathy and clarity. In small group settings, encourage other staff and residents to share their own strategies and specifically on how they cope with these experiences. Additionally, residents can practice different approaches to delivering bad news in role-playing exercises.

Recommended Reading

1. Baile WF, Buckman R, Lenzi R, et al. SPIKES-A six-step protocol for delivering bad news: application to the patient with cancer. Oncologist. 2000;5(4):302-11. PMID 10964998
2. Lee HR, Yi SY. Delivering bad news to a patient: a survey of residents and fellows on attitude and awareness. Korean J Med Educ. 2013 Dec;25(4):317-25. PMID 25804966
3. Monden KR, Gentry L, Cox TR. Delivering bad news to patients. Proc (Bayl Univ Med Cent). 2016 Jan;29(1):101-2. PMID 26722188
4. Narayanan V, Bista B, Koshy C. 'BREAKS' Protocol for Breaking Bad News. Indian J Palliat Care. 2010 May;16(2):61-5. PMID 21811349

Additional Resources

- Rosenbaum ME, Ferguson KJ, Lobas JG. Teaching medical students and residents skills for delivering bad news: a review of strategies. Acad Med. 2004 Feb;79(2):107-17. PMID 14744709

Clinical Care Series

13. Dealing with Difficult Patients

Objective

Provide residents with ways to address and deal with difficult patients in a professional manner.

Background Information

"Difficult patients" encompass a broad range of patient profiles. These patients may include people with drug seeking behavior, who lie, who exhibit personality conflict, and with psychosocial issues. Such patients can negatively impact not only the wellbeing of residents, but also one's ability to accurately diagnose conditions and process clinical findings [2]. Physicians pursue a medical career to help people and make a difference, but sometimes these patients make providers question whether they are indeed making a positive difference in the community. Being aware of triggers that one to be frustrated or irritated is the first step to continued empathy and motivation to help such patients. Once one's triggers are identified, the next step is to having preformed responses and techniques to help to diffuse intense situations and impasses. Some techniques include nonjudgmental listening, patience, tolerance, and empathy [3].

Recommended Approach

Start the large group session by having everyone privately write down specific patient attributes that frustrate, irritate, or annoy them. Share these in a group discussion. After identifying one's triggers, the second half of the session should focus on defusing and coping strategies. Encourage residents and attending physicians to share how they have successfully and unsuccessfully dealt with difficult patients. Conclude the session by collectively generating several preformed scripts for residents to use whenever they encounter challenging patients.

For example, in a patient that has multiple encounters documented as concerning for drug-seeking behavior, you might say, *"I cannot prescribe you any narcotic medications because you have received multiple prescriptions in the past. I am concerned that you may be developing a dependence."*

Recommended Reading

1. Buxton D. Why Are Patients Difficult for Staff? J Palliat Med. 2017 May 25. PMID 28541769
2. Mamede S, Van Gog T, Schuit SC, et al. Why patients' disruptive behaviours impair diagnostic reasoning: a randomised experiment. BMJ Qual Saf. 2017 Jan;26(1):13-18. PMID 26951796
3. Sandikci KB, Üstü Y, Sandikci MM, et al. Attitudes and behaviors of physicians in dealing with difficult patients and relatives: a cross-sectional study in two training and research hospitals. Turk J Med Sci. 2017 Feb 27;47(1):222-233. PMID 28263494

Additional Resources

- Mirza D. The FRAYED Consultation Model for Doctors Dealing with Unreasonable Demands from Difficult Patients. Better Doctor Training Ltd, 2016. [http://amzn.to/2fladIH]

Clinical Care Series

14. Dealing with Difficult Consultants and Staff

Objective

Provide residents with strategies to communicate effectively with behaviorally challenging consultants and staff members.

Background Information

Some of the most frustrating aspects of emergency medicine can be working with difficult consultants or other staff members. Examples include when the hospitalist does not want think hospital admission is warranted for your patient, a consultant yells at you for your management of a patient, or a nurse is extremely slow in administering an urgent medication to your patient despite your conveying the urgency of the matter. These negative emotions and interactions can be demoralizing, taint one's entire ED shift, and negatively impact one's wellbeing. Being prepared with a pre-conceived script or approach can help defuse these challenging moments in a constructive and healthy manner.

Recommended Approach

In a large group setting, discuss how dealing with difficult consultants and staff members are an unfortunate part of the emergency department culture. In a large or small group setting, have residents and faculty members discuss challenging interactions with colleagues and identify common overt and underlying themes. As a group exercise, collectively create professional phrases, scripts, and approaches to deal with these situations when they arise.

Recommended Reading

1. Andrew LB. Conflict management, prevention, and resolution in medical settings. Physician Exec. 1999 Jul-Aug;25(4):38-42. PMID 10557483
2. Grant W. How to Turn Conflict into Cooperation. Collins & Brown Publishing, 2007. [http://amzn.to/2wlK5Bz]
3. Lowes R. Taming the disruptive doctor. Med Econ. 1998 Oct 5;75(19):67-8, 73-4, 77-80. PMID 10185541
4. Pfifferling JH. The disruptive physician. A quality of professional life factor. Physician Exec. 1999 Mar-Apr;25(2):56-61. PMID 10351731

Clinical Care Series

15. Debriefing Traumatic Events in the Emergency Department

Objective

Delineate the importance of and provide effective strategies on debriefing after significant clinical events in the emergency department.

Background Information

Debriefing after significant clinical events is a common practice in emergency departments. Many departments and programs find them to be useful tools for education, as well as improving the system processes that serve patients at critical moments. Many look to the physician as the team leader to convene and conduct the debriefing; however, effective team debriefing is difficult to do, and most physicians receive no formal training on this.

Recommended Approach

In a flipped classroom model, distribute the debriefing journal article by Kessler et al. [1] to residents in order to provide a basic framework on debriefing. This article includes a basic how-to guide as well as a discussion of the benefits and common pitfalls encountered in conducting a debriefing session. In a large group setting, discuss the article and have residents and faculty reflect on their experiences either participating or conducting a debriefing session. At the conclusion, consider implementing some/all of the article's recommendations in the emergency department.

Recommended Reading

1. Kessler DO, Cheng A, Mullan PC. Debriefing in the emergency department after clinical events: a practical guide. Ann Emerg Med. 2015 Jun;65(6):690-8. PMID 25455910

Additional Resources

- Nadir NA, Bentley S, Papanagnou D, et al. Characteristics of Real-Time, Non-Critical Incident Debriefing Practices in the Emergency Department. West J Emerg Med. 2017 Jan;18(1):146-151. PMID 28116028

16. Wellness in the Workplace

Objective

Provide strategies to achieve wellness while at work and explain what factors threaten workplace wellness.

Background Information

The quality of one's work environment can affect one's well-being. Many aspects of the chaotic emergency department workplace can affect resident wellness including the following:

- Safety from physically or verbally violent patients
- Frequent interruptions and need for constant task-switching
- Alarm fatigue
- The electronic medical record
- Few or rare breaks to eat a meal or use the restroom
- Workplace spacing and design
- Emotionally challenging cases
- Suboptimal camaraderie with nurses or ancillary staff

These factors can affect a physician's attitude, mood, concentration, patient care, and ultimately medical decision-making.

Recommended Approach

This module can be done in a large or small group setting. For a larger lecture, a panel 4-5 of attendings, fellows, or senior residents can lead the discussion. For small groups, one assigned group leader should lead the discussion.

Start by introducing the subject and what can affect workplace wellbeing. The first goal of this module is to provide awareness of the potential threats to physician wellness in the workplace, and the effect it can have on a physician's attitude, mood, concentration, patient care, and medical decision-making. The second goal is to identify solutions to these threats.

One suggested topic to review is the misperception that physicians should not take a break from the non-stop work in the emergency department. There is a culture that taking time to eat, drink, or to use the restroom is a sign of weakness and detracts from patient care. There must be a culture of acceptance that physicians should take breaks in order to continue working at peak performance levels.

Recommended Reading

1. Wellness in the Workplace- An Information Paper From the ACEP Well-Being Committee [PDF]. American College of Emergency Physicians website. https://www.acep.org/Physician-Resources/Work-Life-Balance/Wellness/Wellness-in-the-Workplace/. Accessed August 6, 2017.
2. Manfredi RA. One-Minute Stress-Busters. American College of Emergency Physicians website. https://www.acep.org/Physician-Resources/Work-Life-Balance/Wellness/One-Minute-Stress-Busters/. Accessed August 6, 2017.
3. Benzoni T, Manfredi RA. How can we cope with the stresses the EMR Causes? American College of Emergency Physicians website. https://www.acep.org/Physician-Resources/Work-Life-Balance/Wellness/How-Can-We-Cope-With-the-Stresses-the-EMR-Causes-/. Accessed August 6, 2017.
4. Gates DM, Ross CS, McQueen L. Violence against emergency department workers. J Emerg Med. 2006 Oct;31(3):331-7. PMID: 16982376
5. Hospitals: Leading the way in workplace wellness. An initial report of workplace wellness practices in hospitals using workhealthy America. Trust for America's Health website. http://healthyamericans.org/health-issues/wp-content/uploads/2013/09/CDC-Report-Final-9.24.13.pdf. July 2013. Accessed August 6, 2017.
6. Schulte B. Your Sweet Spot: How to become more productive while actually working less. The Washington Post. https://www.washingtonpost.com/news/inspired-life/wp/2015/03/10/your-sweet-spot-how-to-become-more-productive-while-actually-working-less/?utm_term=.1b1ee4d7d86e. March 10, 2015. Accessed August 6, 2017.

17. Dealing with Medical Errors and Shame

Objective

Discuss the reality of medical errors in emergency medicine and how to effectively cope with these errors.

Background Information

Medical errors resulting in patient harm are an unfortunate reality of practicing medicine especially in a specialty like emergency medicine where the stakes are high and information regarding the patient's condition is often limited. These mistakes can lead to feelings of provider inadequacy, shame, and guilt, which can have significant negative downstream effects on overall wellness. The stress of these events often are augmented and become even more overwhelming when peer review committees and hospital risk management are involved.

Recommended Approach

The goal of this module is to normalize discussing a resident's thoughts and attitudes following an event revolving around a medical error. In a large group setting, have a previously-identified faculty member or senior resident volunteer recount a time when they made a medical error that resulted in harm. Have him/her discuss how s/he dealt with any feelings of guilt or shame from the error and lessons learned.

Then in smaller groups, have the residents discuss how they have or would deal with medical errors. Reconvene in a large group to share ideas and experiences. Provide departmental and institutional resources for the residents to access when coping with medical errors.

Recommended Reading

1. Fischer MA, Mazor KM, Baril J, et al. Learning from mistakes. Factors that influence how students and residents learn from medical errors. J Gen Intern Med. 2006 May;21(5):419-23. PMID 16704381
2. Goldman B. Doctors Make Mistakes. Can We Talk About That? TED website. https://www.ted.com/talks/brian_goldman_doctors_make_mistakes_can_we_talk_about_that#t-1143506. November 2011. Accessed August 6, 2017.
3. Hoffman J. Physician's coping after a medical error. KevinMD.com website. http://www.kevinmd.com/blog/2011/05/physicians-coping-medical-error.html. May 10, 2011. Accessed August 6, 2017.
4. Shapiro J. Brigham and Women's Hospital Center for Professionalism and Peer Support. Coping and Recovering After a Medical Error. http://www.brighamandwomens.org/Medical_Professionals/career/CPPS/Documents/Coping_Recovery_mederror_07_2014.pdf. 2014. Accessed August 6, 2017.

Additional Resources

1. Brigham and Women's Hospital Center for Professionalism and Peer Support. Coping and Recovering After a Medical Error. http://www.brighamandwomens.org/medical_professionals/career/cpps/default.aspx. September 18, 2015. Accessed August 6, 2017.
2. Cedfeldt AS, Desai SS. Helping Residents Cope with Bad Outcomes. Society of General Internal Medicine website. http://impak.sgim.org/userfiles/file/AMHandouts/AM08/WB06%20Andrea%20Cedfeldt.pdf Accessed August 6, 2017.
3. Medically Induced Trauma Support Services website. http://www.mitss.org/clinicians_home.html. Accessed August 6, 2017.
